# Only 9% of mothers have eight and more ANC visit in 14 sub-saharan African countries; evidence from the most recent DHS 2018–2023: a multilevel analysis

**DOI:** 10.1186/s12889-024-19145-x

**Published:** 2024-06-19

**Authors:** Kaleb Assegid Demissie, Melak Jejaw, Befikir Gezahegn Wondimu, Yekunuamlak Teshome Mersha, Eyuale Sitotaw Demsash, Samuel Getachew Dessie, Asteway Gashaw Teshome, Demiss Mulatu Geberu, Misganaw Guadie Tiruneh

**Affiliations:** 1 Department of Health Systems and Policy, Institute of Public Health, Collage of Medicine and Health Science, University of Gondar, Gondar, Ethiopia; 2 Department of Obstetrics and Gynecology, Collage of Medicine and Health Science, University of Gondar, Gondar, Ethiopia; 3 Department of Anatomic Pathology, Collage of Medicine and Health Science, University of Gondar, Gondar, Ethiopia; 4 Department of Surgery, Collage of Medicine and Health Science, University of Gondar, Gondar, Ethiopia; 5 Department of Internal Medicine, Collage of Medicine and Health Science, University of Gondar, Gondar, Ethiopia

**Keywords:** ANC, SSA, Eight and more visits, WHO

## Abstract

**Background:**

The world health organization’s global health observatory defines maternal mortality as annual number of female deaths, regardless of the period or location of the pregnancy, from any cause related to or caused by pregnancy or its management (aside from accidental or incidental causes) during pregnancy and childbirth or within 42 days of termination of pregnancy and an estimated 287 000 women worldwide passed away from maternal causes between 2016 and 2020, that works out to be about 800 deaths per day or about one every two minutes.

**Method:**

The most recent 2018–2023 DHS data set of 14 SSA countries was used a total of 89,489 weighted mothers who gave at list one live birth 3 years preceding the survey were included, a multilevel analysis was conducted. In the bi-variable analysis variables with p-value ≤ 0.20 were included in the multivariable analysis, and in the multivariable analysis, variables with p-value less than ≤ 0.05 were considered to be significant factors associated with having eight and more ANC visits.

**Result:**

The magnitude of having eight and more ANC visits in 14 sub-Saharan African countries was 8.9% (95% CI: 8.76–9.13) ranging from 3.66% (95% CI: 3.54–3.79) in Gabon to 18.92% (95% CI: 18.67–19.17) in Nigeria. The multilevel analysis shows that maternal age (40–44, AOR;2.09, 95%CI: 1.75–2.53), maternal occupational status (AOR;1.14, 95%CI; 1.07–1.22), maternal educational level (secondary and above, AOR;1.26, 95%CI; 1.16–1.38), wealth status(AOR;1.65, 95%CI; 1.50–1.82), media exposure (AOR;1.20, 95%CI; 1.11–1.31), pregnancy intention (AOR;1.12, 95%CI; 1.05–1.20), ever had terminated pregnancy (AOR;1.16 95%CI; 1.07–1.25), timely initiation of first ANC visit (AOR;4.79, 95%CI; 4.49–5.10), empowerment on respondents health care (AOR;1.43, 95%CI; 1.30–1.56), urban place of residence (AOR;1.33, 95%CI; 1.22–1.44) were factors highly influencing the utilization of AN. On the other hand higher birth order (AOR;0.54, 95%CI; 0.53–0.66), not using contraceptive (AOR;0.80, 95%CI; 0.75–0.86) and survey year (AOR;0.47, 95%CI; 0.34–0.65) were factors negatively associated with having eight and more ANC visits.

**Conclusion:**

In the 14 SSA included in this study, there is low adherence to WHO guidelines of eight and more ANC visits. Being educated, having jobs, getting access to media being from rural residence and rich wealth group contribute to having eight and more ANC visits, so we highly recommend policy implementers to advocate this practices.

## Background

The world health organization’s global health observatory defines maternal mortality as annual number of female deaths, regardless of the period or location of the pregnancy, from any cause related to or caused by pregnancy or its management (aside from accidental or incidental causes) during pregnancy and childbirth or within 42 days of termination of pregnancy [1]. An estimated 287 000 women worldwide passed away from maternal causes between 2016 and 2020, that works out to about 800 deaths per day or about one every two minutes [2]. When it comes to the case of Africa significant decline in maternal mortality were seen in every region except the sub-Saharan part. with the south experiencing the biggest drops and the north African region is getting near the UN’s sustainable development goals but the remaining Sub-Saharan Africa are still a long way from meeting the targets [3]. the global MMR has shown a reduction through the years, a decrease in 34.3% from the past 20 years worldwide [2].

The likelihood of impoverished women in rural areas receiving quality healthcare is lowest [4]. This is particularly true for SDG areas like Sub-Saharan Africa and Southern Asia that have comparatively low numbers of professionals in the health care industry. and the presence of a trained midwife, doctor, or nurse is beneficial for 99% of births in the majority of high-income and upper-middle-income countries and only 68% of low-income and 78% of lower-middle-income nations receive assistance from such qualified medical professionals [5, 6].

The opportunity for crucial health tasks like illness prevention, screening, diagnosis, as well as health promotion and mainly reduction of maternal mortality is made possible by adequate and timely ANC visit and it is also essential medical tool that lowers the risk of complications from pregnancy, preterm labor, and stillbirths [7–11].

According to studies in low- and middle-income countries (LMICs) the majority of women more than 80% of them receive at least one ANC visit, only 49.3% receive four to seven visits, and only 11.3% receive eight visits [12]. In LMICs, the chance of health facility-based delivery and early PNC utilization was considerably raised up by ANC8 + visits [13]. Hence increasing the frequency of ANC visit will improve health outcomes, provide better pregnancy outcome, reduce maternal mortality and stillbirths, build trust with healthcare providers [14–17]. This in general will increase the existing low ANC attendance. Also since effectiveness of ANC8 + contact has been proven [13]. countries that are still in ANC4 + should scale-up acceptability of 8-ANC contacts or more, Some countries are adjusting their policies based on recent information that suggests eight or more prenatal care contacts could lower the number of perinatal mortality by up to eight per 1000 births, which would benefit both the mother and the unborn child [10, 18].

Different data’s show what factors are associated with ANC visit for example studies conducted in different parts of Africa [19–23] showed that maternal educational status, media exposure, wealth status, parity and marital status had association with ANC utilization. Also, other studies conducted in sub-Saharan countries [24–28] shows that maternal educational status, maternal occupation, media exposure, access to health care, contraceptive use, pregnancy wanted and place of residence were factors associated with ANC visit.

Despite the presence different studies on ANC utilization in sub-Saharan countries our study aims to address the prevalence and associated factors of ANC utilization in 14 Sub-Saharan countries from two new points of views. One being the data set we used was the most recent form 2018–2023 most of the previous studies used old data sets ranging from 2006 to 2016. And two we used the recent WHO recommendation [10] of eight and above visits which was launched in 2016 to determine our outcome variable, given the fact that previous studies recommend future researches to consider the new WHO recommendation and also the DHS-7 guideline recommends future survey to adopt this indicator, So this study aims to understand if countries in Sub-Saharan African region have been implementing this new indicator, and what factors in individual and community level affects the outcome.

## Method and material

### Study area and data source

The sub-Sahara region is made up of all African nations and territories that are situated entirely or partially south of the Sahara and more than 500 million women’s with almost half of them being in the reproductive age group (15–49) live in this region [29]. Our study used the most recent Demographic and Health Survey (DHS) data of 14 sub-Saharan countries collected using a cross-sectional study design from 2018 to 19 and 2023.

The DHS Cross-sectional surveys were used to gather data from women of reproductive age (15–49 years old) on health and other related topics. by giving an explanation of the study on the DHS official website https://dhsprogram.com/data/available-datasets.cfm permission to use the DHS data was acquired. In the DHS surveys, a two-stage stratified sampling method was used to select the sample. Every nation was split up into groups. Enumeration areas (EAs) were chosen for each cluster in the first stage, and a household listing exercise was carried out in each of the chosen EAs. The households on the list served as the foundation for choosing the households and households from each enumeration area were chosen for the second stage. Women between the ages of 15 and 49 who were defacto in each chosen home were chosen and interviews were conducted in person by the fieldworkers. In our study a total of 89,489 weighted women who gave birth in the last three years preceding the surveys were included. Number of women per country and survey year are shown in (Table 1).

**Table 1:** Country, survey year and weighted sample size for the 14 subs Saharan countries

Country	Survey year	Weighted sample size
Burkina Faso	2021	6,332
Cameron	2018	10,403
Coat’ d’ivoire	2021	5,206
Gabon	2019	3,223
Ghana	2022	4,653
Gambia	2019	4,197
Kenya	2022	9,362
Nigeria	2018	17,465
Rwanda	2019–2020	4,598
Sierra Leone	2019	5,432
Senegal	2019	3,210
Tanzania	2022	5,816
Zambia	2018	5,480
Guini	2018	4,112

### Dependent variable

Outcome variable in this study is antenatal care utilization which was measured as not utilized and utilized, and respondents who visited ANC service < 8 times were categorized as not utilized and those who visited ANC service > or = 8 times were categorized as utilized this classification was based on the new WHO recommendation [10].

#### Independent variables

Individual level variables such as maternal age (15–19, 20–24, 25–29, 30–34, 35–39, 40–44, 45–49), maternal educational status (No education, primary, secondary and above), marital status (Not married, married/living with a partner), wealth index (Poor, middle, rich), media exposure (No, yes), respondents’ occupational status (Not working, working), pregnancy intension (intended, un-intended), contraceptive use (user, not user), ever had terminated pregnancy (yes, no), access to health care (no problem, problem) decision on respondents health care (Alone, both, husband alone, others) timely initiation of ANC visit and birth order (1, 2–4, + 5) and community level variables such as place of residence (urban, rural), community level education (yes, no), community level wealth status (yes, no), community level media exposure (yes, no) and Survey year (2018–2020 and 2021–2023) community level variables like community level education, community level wealth status and community level media exposure were created from the individual variables using proportion by adding the individual-level variables in to clusters.

### Data analysis

Stata version-17 software was used for analysis, descriptive statistics was conducted using cross tabulation and by calculating frequency and percentage to show how individual and community level characteristics were distributed and a multilevel binary logistics regression was used to assess if individual and community level factors affect ANC utilization. In the bi-variable regression analysis, variables with a p-value ≤ 0.2 were considered in the multivariable analysis and the Adjusted Odds Ratio (AOR) with a 95% Confidence Interval (CI) at p-value < 0.05 in the multivariable multilevel analysis was used to declare statistical significance. For the data to be representative and to account the probability of unequal selection between clusters both descriptive and analytic analysis were carried out after the weighting of data using individual weights for women (v005) then we generated individual weight by dividing to 100,000. And since the nature of the DHS data is hierarchical meaning there are some variables snuggled within other variables. In order to consider between cluster variability, we created four models the null model with only the dependent variable, model 1 which includes the dependent variable and individual-level variables, model two which includes the dependent variable with the community level variables and model for the dependent variable with both the individual and community level variables. To assess the presence of clustering and to check the appropriateness of multilevel logistic regression we calculated Intraclass Correlation Coefficient (ICC), proportional change in variance (PCV) and median odds ratio (MOR). The ICC in the empty model is 28.3% which is greater than 5% this indicates that 28.6% of the variability in ANC utilization is explained by between-Clusters differences and 71.7% of the variability is explained by within Clusters. The best fit model was selected by comparing the Akaike Information Criteria (AIC) and Deviance (-2LLH) so the model with lowest Akaike Information Criteria (AIC) and smallest Deviance were selected.

ICC and AIC were calculated by using the Stata command “***estat icc****” and “****estat ic****”* respectively after running the “*melogit command*” for each model.

PCV were calculated for each model, ***PCV= (V***_***null***_*−V*_***M1/M2/M3***_***/V***_***null***_) *100.

Vnull = the variance in the Null model, VM1 = variance in Model one, VM2 = variance in Model two, VM3 = variance in Model three.

MOR was calculated for each model = ***exp(0.95√VA)***.

## Results

### Individual level factors

A total of 89,489 mothers who gave birth in the past three years before the survey and who attend ANC visits for their last pregnancy were included in the study, 23,500 (26.26%) were aged between 20 and 29 age group, around 85.90% (76,870) respondents were either married or living with a partner. 33,638 (37.59%) of them have finished secondary school, 61,669 (68.91%) of the respondents have exposure to media, 29,140 (32.56%) of respondents were Muslims, majority of the respondents 39,068 were from the poor wealth group, 60.82% (54,424) respondents are currently working, 74.71% (66,857) of the pregnancies were intended. 60,091 or (67.15%) of mothers are not contraceptive users, 86.04% of mother had never had a terminated pregnancy, 60.91% of the respondents believed there is no problem in access to health care, 36,883 (41.21%) of respondent’s health care decision were done by the husband only, 57.56% or 46,984 of the respondents had a delayed first ANC visit (Table 2).

**Table 2:** Individual characteristics of study participants in sub-Saharan Africa (*n* = 89,489)

Individual level variables	Frequency	Percentage
**Maternal age**		
15–19	7,270	8.12%
20–24	20,750	23.19%
25–29	23,500	26.26%
30–34	18,488	29,66%
35–39	13,001	14.53%
40–44	5,201	5.81%
45–49	1,279	1.43%
**Maternal education**		
No education	32,084	35.85%
Primary	23,767	26.56%
Secondary+	33,638	37.59%
**Marital status**		
Not married	12,619	14.10%
Married/living with partner	76,870	85.90%
**Religion**		
Muslim	29,140	32.56%
Catholic	25,005	27.94%
Protestant	16,801	18.77%
Traditional/other	18,544	20.72%
**Wealth index**		
Poor	39,068	43.66%
Middle	17,660	19.73%
Rich	32,761	36.61%
**Media exposure**		
No	27,820	31.09%
Yes	61,669	68.91%
**Respondents’ working status**		
Not working	35,065	39.18%
Working	54,424	60.82%
**Pregnancy intention**		
Un-intended	22,632	25.29%
Intended	66,857	74.71
**Contraceptive use**		
Not user	60,091	67.15%
User	29,398	32.85%
**Ever had terminated pregnancy**		
No	76,998	86.04%
Yes	12, 490	13.96%
**Access to health care**		
No problem	54,511	60.91%
Problem	34,978	39.09%
**Who decides on respondents health care**		
Alone	12,739	14.24%
Both	26,786	29.93%
Husband alone	36,883	41.21%
Others	13,080	14.62%
**Birth order**		
1	20,602	23.02%d
2–4	44,522	49.75%
+ 5	24,364	27.23%
**Timely initiation of ANC visit**		
Timely	34,636	42.44%
Delayed	46,984	57.56%

### Community level factors

From the study participants 54,611 (59.91%) of the mothers were from rural area, almost half of the respondents 51.96% were from a community that has high proportion of people being highly educated, 52.6% of women were from communities with high proportion of media exposure (Table 3).

**Table 3:** Community level characteristics of study participants (*n* = 89,489)

Community level variables	Frequency	Percentage
**Residence**		
Urban	35,878	40.09%
Rural	53,611	59.91%
**Community level education**		
Low	42,993	48.04%
High	46,495	51.96%
**Community level media exposure**		
Low	42,407	47.39%
High	47,082	52.61%
**Community level wealth status**		
Low	42,447	47.43%
High	47,042	52.57
**Survey year**		
2018–2020	58,120	63.46
2021–2023	31,369	36.54

### Prevalence of ANC utilization in 14 SSA countries

The overall prevalence of eight and more ANC visits in 14 SSA countries was 8.9% (95% CI: 8.76–9.13) ranging from 3.66% (95% CI: 3.54–3.79) in Gabon to 18.92% (95% CI: 18.67–19.17) in Nigeria.

### Random effects

The null model illustrated a variation in the likelihood of eight and more ANC visits in sub–Saharan African countries between clusters indicating that 28.3% of the variation in eight and more ANC visits in 14 SSA was attributed to Intra-Class Correlation variation (ICC = 0.283). for the individual level variables only in model 1 the between-cluster variation dropped to 25.6% (ICC = 0.256) and in the community-level variables which is model 2, the ICC decreased to 23% (ICC = 0.23). In contrast, in the third model which contains both the individual and community level variables the ICC, further decreased to 18.5% (ICC = 0.185). Moreover, the final model had the lowest MOR value (2.26) indicating that the odds of eight and more ANC visit was 2.26 times higher when we move from low ANC visit areas to higher areas. Also, as the PCV indicates for the last model that is model 3 (PCV = 0.43) 43.0% of the variation in eight or more ANC visit across clusters was explained by both individual and community-level factors. Again, the last model (Model 3) has the lowest (36,852.54) deviance and lowest (36,944.54) AIC. So, model 3 was used to identify factors associated with eight and more ANC visits in 14 SSA. (Table 4)

**Table 4:** Random effect

Random effect	Null model	Model 1	Model 2	Model 3
LLH	-26299.398	-22594.82	-21080.093	-18426.27
Variance	1.30	1.13	0.98	0.74
ICC	28.3	25.6	23	18.5
PCV	Reference.	13	24.6	43.0
AIC	52602.8	45245.64	42200.14	36944.54
MOR	2.94	2.73	2.55	2.26

### Individual and community level factors associated with eight and more ANV visits

#### Model 1 (Individual level factors only)

In the model accounted for individual level variables only (model 1), maternal age, maternal occupational status, maternal educational level, wealth status, media exposure, pregnancy intention, ever had terminated pregnancy, timely initiation of first ANC visit, empowerment on respondents’ health care, access to health care, and birth order. Were variables associated with eight and more ANC visit (Table 5).

**Table 5:** Multilevel logistic regression analysis to determine factors associated with ANC utilization in reproductive-age women in 14 SSA countries

Variables	*P*-value in bivariable analysis	Null model	Model 1 AOR (95%CI), *P*-value	Model 2 AOR (95%CI), *P*-value	Model 3 AOR (95%CI), *P*-value
**Maternal age**	0.000				
15–19			1		1
20–24			1.04, (0.91–1.17)		1.13, (0.99–1.29)
25–29			1.19, (1.05–1.36) **0.006**		1.27, (1.10–1.46) **0.001**
30–34			1.45, (1.26–1.66) **0.000**		1.61, (1.39–1.87) **0.000**
35–39			1.60, (1.38–1.85) **0.000**		1.76, (1.51–2.07) **0.000**
40–44			1.68, (1.41–1.99) **0.000**		2.09, (1.74–2.53) **0.000**
45–49			1.89, (1.46–2.45) **0.000**		1.75, (1.31–2.32) **0.000**
**Maternal education**	0.000				
No education			1		1
Primary			0.80, (0.73–0.87) **0.000**		1.20, (1.09–1.32), **0.000**
Secondary+			1.82, (1.68–1.97) **0.000**		1.26, (1.16–1.38), **0.000**
**Marital status**	0.000				
Not married			1		1
Married/living with partner			0.86, (0.54–1.35)		1.07, (0.65–1.77)
**Religion**	0.000				
Muslim			0.66, (0.50–0.88)		1.04, (0.92–1.10)
Catholic			1		1
Protestant			0.88, (0.68–1.13)		0.71, (0.64–1.79)
Traditional/others			1.01, (0.41–2.49)		0.95, (0.84–1.08)
**Wealth status**	0.000				
Poor			1		1
Middel			1.34, (1.24–1.46) **0.000**		1.17, (1.07–1.28), **0.000**
Rich			1.79, (1.66–1.93) **0.000**		1.65, (1.50–1.82), **0.000**
**Media exposure**	0.000				
No			1		1
Yes			0.98, (0.91–1.95)		1.20,(1.11–1.31), **0.000**
**Respondents’ occupation**	0.000				
Not working			1		1
Working			1.73, (1.63–1.83) **0.000**		1.14, (1.07–1.22), **0.000**
**Pregnancy intention**	0.000				
Un-intended			1		1
Intended			1.17, (1.09–1.24) **0.000**		1.12, (1.05–1.20), **0.001**
**Contraceptive use**	0.000				
Not user			1.33, (1.26–1.41) **0.000**		0.80, (0.75–0.86), **0.000**
User			1		1
**Ever had terminated pregnancy**	0.000				
No			1		1
Yes			1.19, (1.11–1.27), **0.000**		1.16, (1.07–1.25), **0.000**
**Timely initiation**	0.000				
Timely			3.79, (3.58–4.01), **0.000**		4.79, (4.49–5.10), **0.000**
Delayed			1		
**Decision on health care**	0.000				
Respondent alone			1.09, (1.00-1.18), **0.028**		1.43, (1.30–1.56), **0.000**
Both			1.17, (1.09–1.25), **0.000**		1.28, (1.19–1.38), **0.000**
Husband alone			1		1
Others			0.85, (0.54–1.33)		1.31, (0.80–2.16)
**Access to health care**	0.000				
No problem			1.20, (1.14–1.27), **0.000**		0.97, (0.90–1.03)
Problem			1		1
**Birth order**	0.000				
1			1		1
2–4			0.90, (0.84–0.97), **0.000**		0.84, (0.78–0.91), **0.000**
+ 5			0.73,(0.66–0.81), **0.000**		0.59, (0.53–0.66), **0.000**
**Community level variables**					
**Residence**	0.000				
Urban				2.28, (2.14–2.44), **0.000**	1.33, (1.22–1.44), **0.000**
Rural				1	1
**Survey year**	0.000				
2018–2020				1	1
2021–2023				0.41, (0.30–0.56), **0.000**	0.47, (0.34–0.65), **0.000**
**Community level education**	0.000				
Low				1	1
High				2.42, (2.05–2.87), **0.000**	1.57, (1.35–1.84), **0.000**
**Community level media exposure**	0.000				
Low				1	1
High				2.19, (1.86–2.59), **0.000**	1.70, (1.45–1.98), **0.000**
**Community level wealth status**	0.000				
Low				1	1
High				1.03, (0.29–1.20),	0.89, (0.77–1.03)

#### Model 2 (Community level factors only)

Where as in the model accounted for community level variables only, place of residence, community level education, community level media exposure and survey year were factors associated with eight and more ANC visit (Table 5).

#### Model 3 (Individual and community level factors)

In the Final model containing both individual and community level factors (model 3) maternal age, maternal occupational status, maternal educational level, wealth status, media exposure, pregnancy intention, ever had terminated pregnancy, timely initiation of first ANC visit, empowerment on respondents’ health care, and birth order, place of residence, community level education, community level media exposure and survey year were factors associated with eight and more ANC visit.

The odds of having eight and more ANC visit was 1.27 (AOR = 1.27; 95% CI: 1.10–1.46), 1.61 (AOR = 1.61; 95% CI: 1.39–1.87), 1.76 (AOR = 1.76; 95% CI: 1.51–2.07), 2.09 (AOR = 2.09; 95% CI: 1.74–2.53) and 1.75(AOR = 1.75; 95% CI: 1.31–2.32) times higher among respondents aged 25–29, 30–34, 35–39, 40–44, and 45–49 years respectively when compared to women aged 15–24 years, The odd of having eight and more ANC visit timely initiation of ANC was 1.20 (AOR = 1.20; 95% CI: 1.09–1.32) and 1.26 (AOR = 1.26; 95% CI: 1.16–1.38) times higher in mothers who had primary and secondary education respectively as compared to those mothers who had no formal education. Women in the middle and richest wealth categories had 1.17 (AOR = 1.17; 95% CI: 1.07–1.28) and 1.65 (AOR = 1.65; 95% CI: 1.50–1.82) higher odds of eight and more ANC visit respectively when compared to the poor women. When it comes to media exposure respondents who has exposure to media has a higher odd of having eight and more ANC visit than those with no media exposure 1.20 (AOR = 1.20; 95% CI: 1.11–1.31). Mothers whose last pregnancy was intended had 1.12 (AOR = 1.12; 95% CI: 1.05–1.20) higher odds of having eight or more ANC visit when compared to who has unintended pregnancy. There is a higher odd of eight and more ANC visit among women who had terminated pregnancy 1.16 (AOR = 1.16; 95% CI: 1.07–1.22) than those with no history of termination. Additionally, the odds of having eight or more ANC visit were 20% (AOR = 0.80; 95% CI: 0.75–0.86) lower for mothers who are not contraceptive users compared to contraceptive user women. Also, the odds of having eight or more ANC visit were 16% (AOR = 0.84; 95%CI: 0.78–0.91) and 41% (AOR = 0.59; 95% CI: 0.53–0.66) lower among women with birth order of between two-four and five and greater than five respectively compare to those women with only one birth order. When it comes to timely initiation of first ANC visit, mothers with timely initiation had 4.79 (AOR = 4.79; 95% CI: 4.49–5.10) times higher odds of having eight or more ANC visit than mothers with delayed initiation. The odds of having eight or more ANC visit were higher among respondents who make their health care decision Alone 1.43 (AOR = 1.43; 95% CI: 1.30–1.56) and with their husbands together 1.28 (AOR = 1.28; 95% CI: 1.19–1.38) compared to respondents whose health care decision was made by their husband alone. A woman who was living in the urban area had 1.33 (AOR = 1.33; 95%CI: 1.22–1.44) higher odds of eight and more ANC visit as compared with a woman who was living in rural areas. Furthermore, women from highly educated communities and communities with high media exposure had higher odds 1.57 (AOR = 1.57; 95% CI: 1.35–1.84) and 1.70 (AOR = 1.70; 95% CI: 1.45–1.98) respectively compared with the counter parts. In addition, respondents who were surveyed from 2021 to 2023 had 57% (AOR = 0.47, 95% CI: (0.34–0.65), lower odds of having eight or more ANC visits compared to women surveyed from 2018 to 2020 (Table 5).

## Discussion

In order to avoid maternal and newborn mortality ANC utilization/visit has a great deal of worth, our study aimed to understand if the new WHO recommendation had been adapted and to analyze individual and community level factors influencing eight and more ANC visits, among women of reproductive age 15–49 years in 14 Sub-Saharan African countries using the most recent DHS data set from 2018 to 2023.

Our study showed that only 8.9% (95% CI: 8.76–9.13**)** of women aged (14–49) had eight and more ANV visit. Ranging from 3.66% (95% CI: 3.54–3.79) in Gabon to 18.92% (95% CI: 18.67–19.17) in Nigeria. Previous study [30] conducted in sub-Saharan African countries showed a 58.53% ANC utilization, this difference might be mainly due to this study used Four and above ANC visits to consider as ANC utilized, while our study used WHOs eight and above recommendation. but our finding is almost similar with the study conducted also in sub-Saharan African countries which shows 6.8% of respondents had eight and more visits [25]. This shows that even though previous studies [25] recommend to develop, adapt and execute new and improved programs and policies to achieve WHOs recent recommendation, countries are not applying the new recommendation of eight and more ANC visits.

Based on the finding of our study there is a strong correlation between a woman’s age and her ability to receive appropriate ANC service, the result showed that a woman is more likely to use prenatal care appropriately the older she gets, this suggest that, in comparison to older women, young women most likely lack experience with pregnancy care. This finding is in line with other studies [31–33]. This could be explained by the fact that women who became pregnant earlier had more life experience and knowledge of the advantages of going to medical facilities.

Our study showed that education levels were significantly associated with eight and more ANC visits among reproductive aged women. Higher education levels associated with higher number of ANC visits this finding agrees with previous studies [23, 25, 27, 30, 34–37] possible explanation might be that educated women will be more aware of the benefits of ANC visits. Additionally, educated women are more likely to be exposed to information regarding maternal health. So, increasing women’s education, household income, and ability to make decisions results in higher Utilization of maternity healthcare services.

This study showed that women with better wealth status (middle, rich) had higher number of antenatal care visit compared to poor wealth index. The observation that poorest women having lower number of ANV visits are supported by some previous studies [27, 38–40]. This can be explained by since mothers who have higher incomes may be able to afford the costs of health services, including transportation, prescription drugs so they may attend ANV visit regularly, but those women from poor wealth status attend ANC clinics occasionally or never attend the clinics at all.

This study showed that women who were exposed to mass media had a higher likelihood of using the recommended ANC utilization of eight and more than women who were not. This may be the result of the mass media’s ability to inform large audiences at once and also helps in changing perspectives regarding maternal health services and their benefits, this finding is supported by other studies as well [7, 41, 42].

Regarding occupation result of our study reviled that women how have jobs were more likely to have eight and more ANV visits compared to mother with no job, this could be the case because well-paid and employed women do not have financial barrier to ANC services, and also increases their decision-making power. Other studies also agree with this finding [25, 43, 44].

When it comes to women with planed pregnancy, the result of this paper shows that they had a higher chance of having eight and more ANC visit than women with unintended pregnancies this result is consistent with similar other studies [45–48]. It goes without saying that if a woman wanted the pregnancy, she would be more willing to seek medical attention, and on the way if there is an early pregnancy diagnosis this would encourage them for early ANC booking and raise the number of ANC visits. This again also implies that, women’s usage of ANC services may have been adversely affected by the lack of a pregnant mindset, which is typical in unplanned or unexpected pregnancies.

Women who had timely initiation of first ANC visit had a higher chance of completing the recommended eight and more ANC visits than those with delayed first initiation, reason for this might be if women start their first visit within 12 weeks of pregnancy, they have higher possibility of attending eight and more ANC visits before they gave birth.

Different from other Studie [49]. conducted another factor associated with eight and more ANC visit was those women who had terminated pregnancy, main and clear reason may be since previous pregnancy terminations may be linked to specific risk factors or health issues that need to be closely watched during subsequent pregnancies women who experience this condition before will have frequent ANC to prevent and control these risks.

In this study consistent with other studies [50, 51]. women who were not current contraceptive users were less likely to have frequent ANC visits than who use family planning, reason might be, it is believed that family planning users have the idea and knowledge about importance of family planning methods, family planning counselling and also importance of ANC visits,

Birth order was another factor associated with eight and more visit, this may be due to the fact that limiting the number of births can both improve maternal health and the use of maternal health services [30]. This result is similar with other findings [52] but other studies showed that birth order was negatively associated with eight or more ANC visits [25]. This difference might be due the time or year of the survey.

Women who had a power to decide about their health care by themselves and with their husband had a higher chance of having eight and more ANC visit than those women whose decision is made for them by their husband alone. This may be due to the fact that using their autonomy women can make decisions about getting healthcare for themselves. Similar studies also showed similar finding [39, 53–55].

Another factor influencing the use of eight and more ANC visit like previous studies [19, 38, 56, 57] is residence. In comparison to rural areas, the rate of having eight and more ANC visit is higher in urban areas. This might be due to urban areas have a greater concentration of infrastructure like health facilities, which cannot be easily available in rural areas. so, this makes it possible for women living in cities to have frequent access to healthcare. Also in addition, like women who have higher education, jobs, and who have access to media women in urban areas have greater access to health-related information than do women in rural areas.

## Conclusion

In the 14 SSA included in this study, there is low adherence to WHO guidelines of eight and more ANC visits. We highly recommended that programs working to increase maternal and child health should improve or may be change their current policies and create new ones in order to achieve the using of WHO-recommended minimum of eight ANC interactions. Also educating women, increasing benefit of prenatal care through mass media and empowering women should be the main focus areas of policies and programs regarding maternal and child health.

### Strength and limitation

Our study used the most recent 2018–2023 DHS data set for the analysis to determine if countries have been implementing the WHOs new indicator eight and more ANC visit and also the study’s conclusions are corroborated by extensive data sets that encompass 14 SSA nations. However, it is impossible to infer a cause-and-effect relationship between the independent and dependent variables because of the cross-sectional nature of the study, and also since this study did not assess the spatial distribution analysis, we couldn’t identify the hot spot areas recommend future researches to identify hotspot area.

## Data Availability

The datasets generated during the study are publicly available from the DHS official website https://dhsprogram.com/data/available-datasets.cfm.

## Abbreviations

ANC: Antenatal care
AOR: Adjusted odds ratio
DHS: Demographic health survey
ICC: Intra class correlation
MOR: Median odds ratio
PCV: Proportional change variance
LLR: Log likelihood ratio
SSA: Sub-Saharan Africa
